# A synthetic shadow dataset of agricultural settings

**DOI:** 10.1016/j.dib.2024.110364

**Published:** 2024-03-23

**Authors:** Mengchen Huang, Ginés García-Mateos, Ruben Fernandez-Beltran

**Affiliations:** Department of Computer Science and Systems, University of Murcia, 30100 Murcia, Spain

**Keywords:** Rendered shadow images, Computer vision, Shadow detection, Deep learning, Agrovoltaic systems, Blender, Human activity recognition

## Abstract

Shadow, a natural phenomenon resulting from the absence of direct lighting, finds diverse real-world applications beyond computer vision, such as studying its effect on photosynthesis in plants and on the reduction of solar energy harvesting through photovoltaic panels. This article presents a dataset comprising 50,000 pairs of photorealistic computer-rendered images along with their corresponding physics-based shadow masks, primarily focused on agricultural settings with human activity in the field. The images are generated by simulating a scene in 3D modeling software to produce a pair of top-down images, consisting of a regular image and an overexposed image achieved by adjusting lighting parameters. Specifically, the strength of the light source representing the sun is increased, and all indirect lighting, including global illumination and light bouncing, is disabled. The resulting overexposed image is later converted into a physically accurate shadow mask with minimal annotation errors through post-processing techniques. This dataset holds promise for future research, serving as a basis for transfer learning or as a benchmark for model evaluation in the realm of shadow-related applications such as shadow detection and removal.

Specifications Table

**Table:** 

Subject	Computer Vision and Pattern Recognition
Specific subject area	Shadow detection for agricultural environment with human activity
Data format	Raw, Filtered
Type of data	Image
Data collection	The data presented in this work was generated using Blender rendering software, where a 3D scene was created from scratch and models sourced from the Internet were incorporated. The rendering process involved the utilization of a NVidia 4090 graphics card and the Cycles renderer, with 1500 samples and a 0.12 adaptive threshold for each image. Additionally, the Intel Open Image Denoiser was employed to reduce rendering time.
Data source location	University of Murcia, Murcia, Spain. Lat/Lon: 38.0237, -1.1740
Data accessibility	Repository name: Hugging Face Data identification number: 10.57967/hf/1652 Direct URL to data: https://www.doi.org/10.57967/hf/1652

## 1 Value of the Data

• Many openly available shadow detection datasets, such as SBU [2] and ISTD [3], exhibit relatively small sample sizes (approximately 5000 for SBU and around 2000 for ISTD). These datasets rely on manual annotations, which may introduce errors. In contrast, the utilization of a synthetic dataset generated from a 3D scene facilitates the creation of highly accurate shadow annotations, addressing challenging lighting conditions such as low light shadows, self-shadowing, and very soft shadows that are difficult to distinguish even for the human eye. This methodology enables the generation of an extensive dataset featuring physically precise shadow masks.
• This dataset holds significant value for researchers engaged in the study of plant and crop health monitoring or the assessment of photovoltaic panel output within agrovoltaic systems, especially when considering motorized photovoltaic panels.
• By harnessing physically accurate ground truth and a substantial volume of data, this dataset proves valuable for benchmarking alternative models. It can also be employed in conjunction with other datasets for transfer learning and fine-tuning purposes.

## 2 Background

In the domain of computer vision, the focal point of research revolves around shadow detection and segmentation, with applications ranging from shadow removal to broader problem-solving in computer vision. Our specific focus lies in scenarios where the absence or presence of shadows is crucial, notably in outdoor settings where direct sunlight impacts image areas. Our research addresses practical challenges, exemplified in the agrovoltaic domain, where motorized photovoltaic panels and cameras are strategically positioned at a height of approximately 3 meters. The objective is to optimize solar radiation for both energy production and crop growth simultaneously. By leveraging shadow detection from the cameras, we aim to fine-tune the positioning of the photovoltaic panels in future iterations, offering a solution with implications beyond image processing, tackling real-world challenges in diverse fields such as solar energy efficiency and crop health monitoring.

## 3 Data Description

**Fig. 1:**
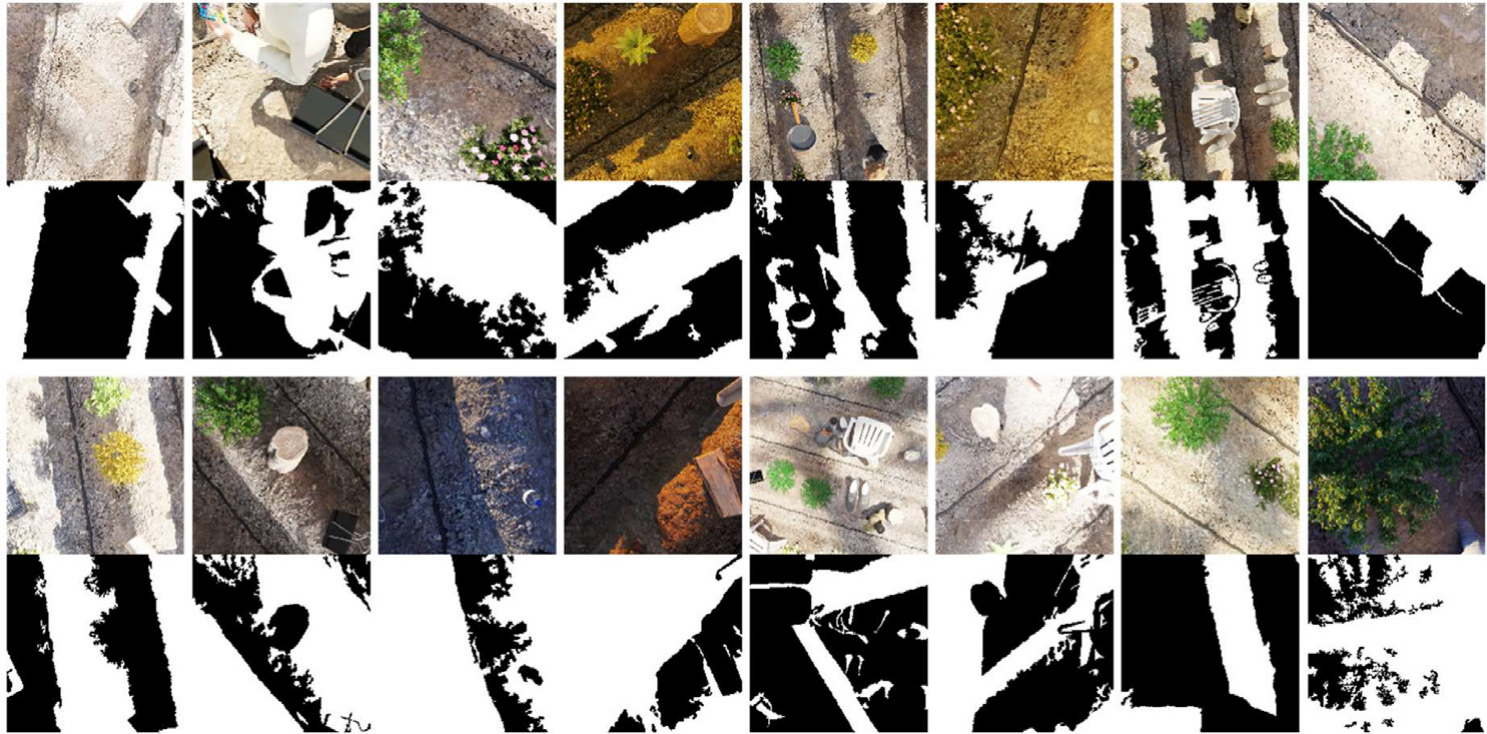
Preview of AgroSegNet.

The AgroSegNet [1], illustrated in Fig. 1, is a shadow detection dataset stored in the Hugging Face Dataset repository. The “default” subset consists of 50,000 pairs of images and their corresponding shadow masks, with a split of 40,000 for training (∼26GB in size) and 10,000 for testing (∼10GB in size). Additionally, a smaller subset, “default-tiny,” is available, comprising 12,500 image pairs, with 10,000 for training and 2,500 for testing.

The dataset can be previewed online on Hugging Face without the need to download the entire collection. Alternatively, it can be accessed directly by downloading the Parquet file or by utilizing Hugging Face's datasets, which allows for loading the dataset with a single line of code, as demonstrated in the README of the data repository.

**Fig. 2:**
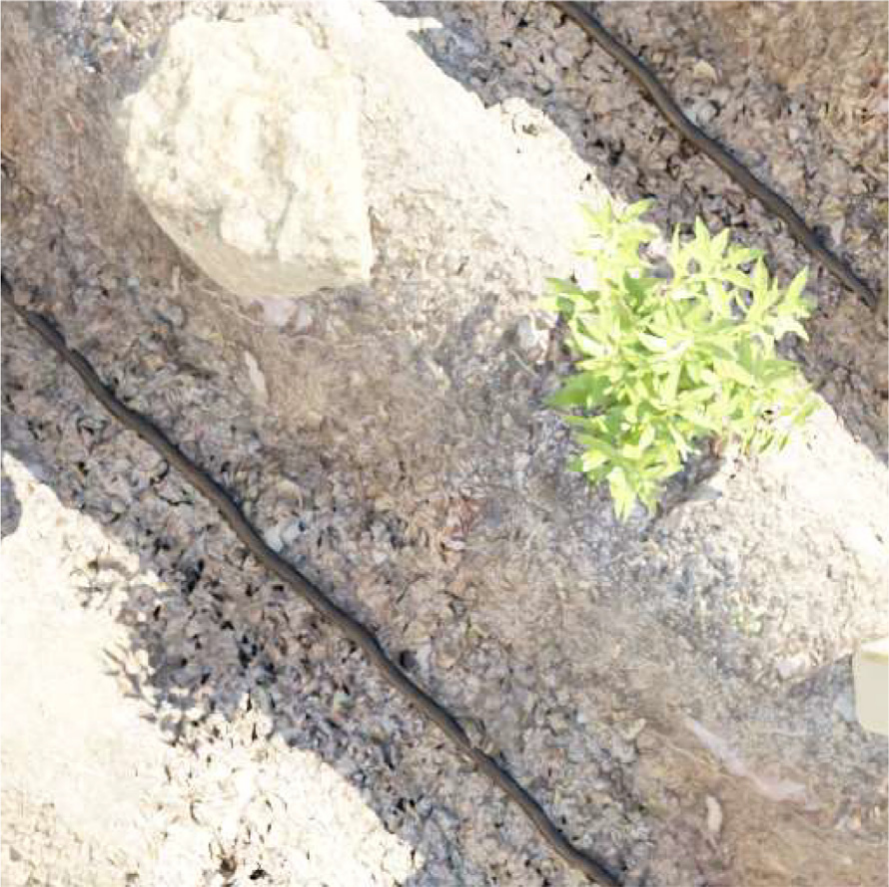
Sample entry from the dataset ‘image’ category.

**Fig. 3:**
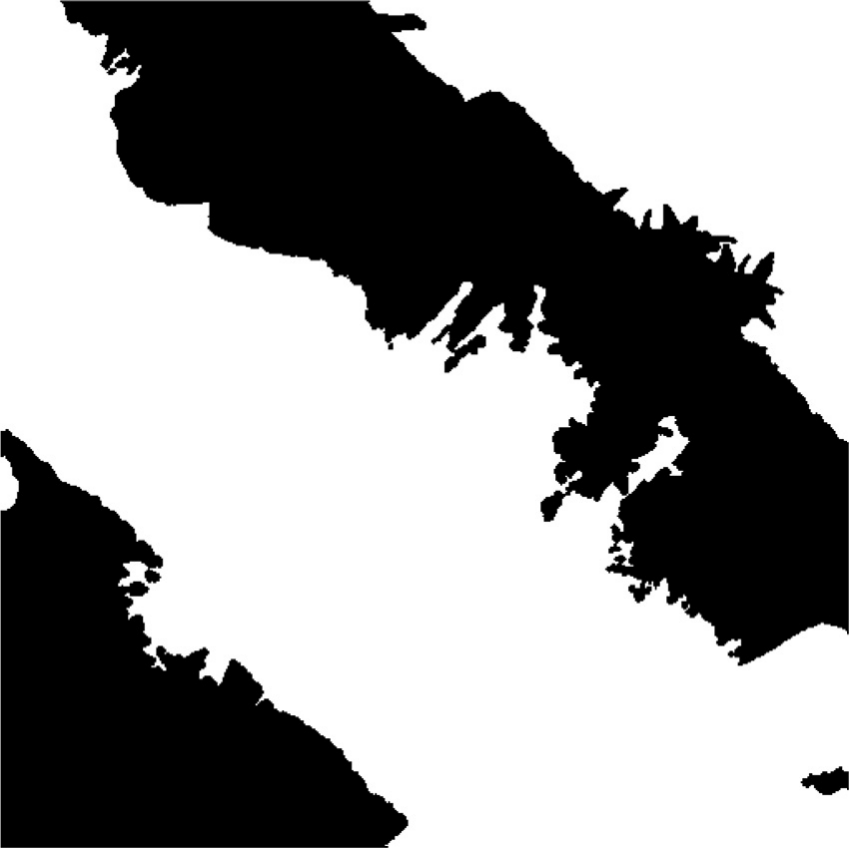
Sample dataset entry in the ‘label’ category depicting a shadow mask.

The dataset follows the structure of a DatasetDict, divided into two subsets: “train” and “test.” Each subset comprises a list of labeled data instances. Each data instance is represented as a dictionary with two key-value pairs: “image,” which corresponds to the image data, as depicted in Fig. 2, and “label,” which corresponds to the associated shadow mask, as illustrated in Fig. 3.

## 4 Experimental Design, Materials and Methods

To acquire the dataset, a 3D scene simulating a plot with strip cropping was meticulously crafted in Blender [4], an open-source 3D modeling software. Several free, textured, and ready to use plant models and random props models acting as distractor were chosen from the internet based on their price, licensing terms and similar artistic style, with the concrete aim of achieving photorealism. These models were incorporated into the scene under licenses that either allow royalty-free usage (permitting commercial use without attribution but prohibiting resale of the asset in its original form) or have no rights reserved (CC0). For texturing the ground, multiple types of ground textures, such as soil, gravel, and mud, were employed. The final ground material was generated procedurally by overlaying these textures, controlled by multiple Perlin noises in a custom shader.

Subsequently, models representing plants and other distractors were instantiated into a grid pattern using Blender's geometry node, a visual node-based method akin to visual programming. To introduce shadows cast by objects outside the camera's view such as cloud or PV panel, a set of objects known as shadow casters was created. These objects, randomly positioned above our downward-facing camera, had the sole function of projecting shadows. They consisted of procedurally instanced simple shapes (e.g., cube, torus, honeycomb, cone, cylinder, and more) without texture and distributed randomly. When combined with sun's strength, these shadow casters simulated various weather conditions, such as cloudiness when many shadows were cast onto the scene or sunshine when there was a lack of shadow caster presence. Additionally, five textured human models with 14 preset poses controlled by a script were introduced to simulate human activity in the field.

For lighting, the Blender add-on “*Sun Position*” was employed, along with the Nishita [5] Sky Texture to simulate real-world natural lighting. This add-on uses physical characteristics to position the sun in the scene based on geographic location, time, and date. In this case, the location is fixed in the northern hemisphere, specifically in Murcia, Spain. The time ranges from 8 am to 6 pm, with altitude randomized from 0 to 300 meters and sun disc size from 0.5 to 0.595 radians. To introduce more lighting color variety, parameters such as air density, dust density, and ozone density are randomized as well.

To generate shadow masks, Blender provides a *shadow catcher* that can capture any shadow cast on an object. However, due to our requirement for self-shadowing (i.e., crops receiving shadows from themselves and others, and casting shadows onto themselves and others), a workaround is employed. Another image is rendered with all light sources disabled, including global illumination from the environment, except for the directional light representing the sun. Indirect lighting is disabled by limiting the maximum bounces to 0, and the sun's strength is increased so that any region affected by directional lighting is overexposed to almost pure white, while the shadow region remains black due to the absence of other light sources.

To facilitate the automation of the rendering process, a script leveraging the Blender API was employed to generate images with a resolution of 512 × 512 pixels. The script utilizes a deterministic seed, derived from the image ID, to introduce randomness into various aspects of the rendering process. Specifically, the camera's transform, ground shader parameters, geometry nodes responsible for plant, distractors and shadow casters distribution, as well as atmospheric parameter used by Nishita, and the sun's position are randomized based on this seed. Refer to Table 1 for a comprehensive breakdown of the randomized parameters and their ranges.

**Table 1:** Summary of 3D objects present in the scene along with their randomized parameter ranges. All random parameters are generated using a continuous uniform distribution.

Plants	7 full textured models
Distractors	21 full textured models
Shadow caster	21 different primitive shapes
Time of day	8am to 6pm
Sun disc size	0.5deg to 0.595deg
Nishita air density	[0-10] Unit
Nishita dust density	[0-10] Unit
Nishita ozone density	[0-10] Unit
Nishita altitude	[0-300] Meters
Camera focal length	[47-53] mm
Sun intensity multiplier	[0.7-2]
Environment light intensity multiplier	[0.2,1.3]

The lighting parameters mentioned earlier are adjusted within the script, and a distinct output name is assigned for each pair of the normal render and the overexposed render, which are subsequently utilized in generating the shadow mask. This approach ensures that each rendering instance is uniquely identified.

**Fig. 4:**
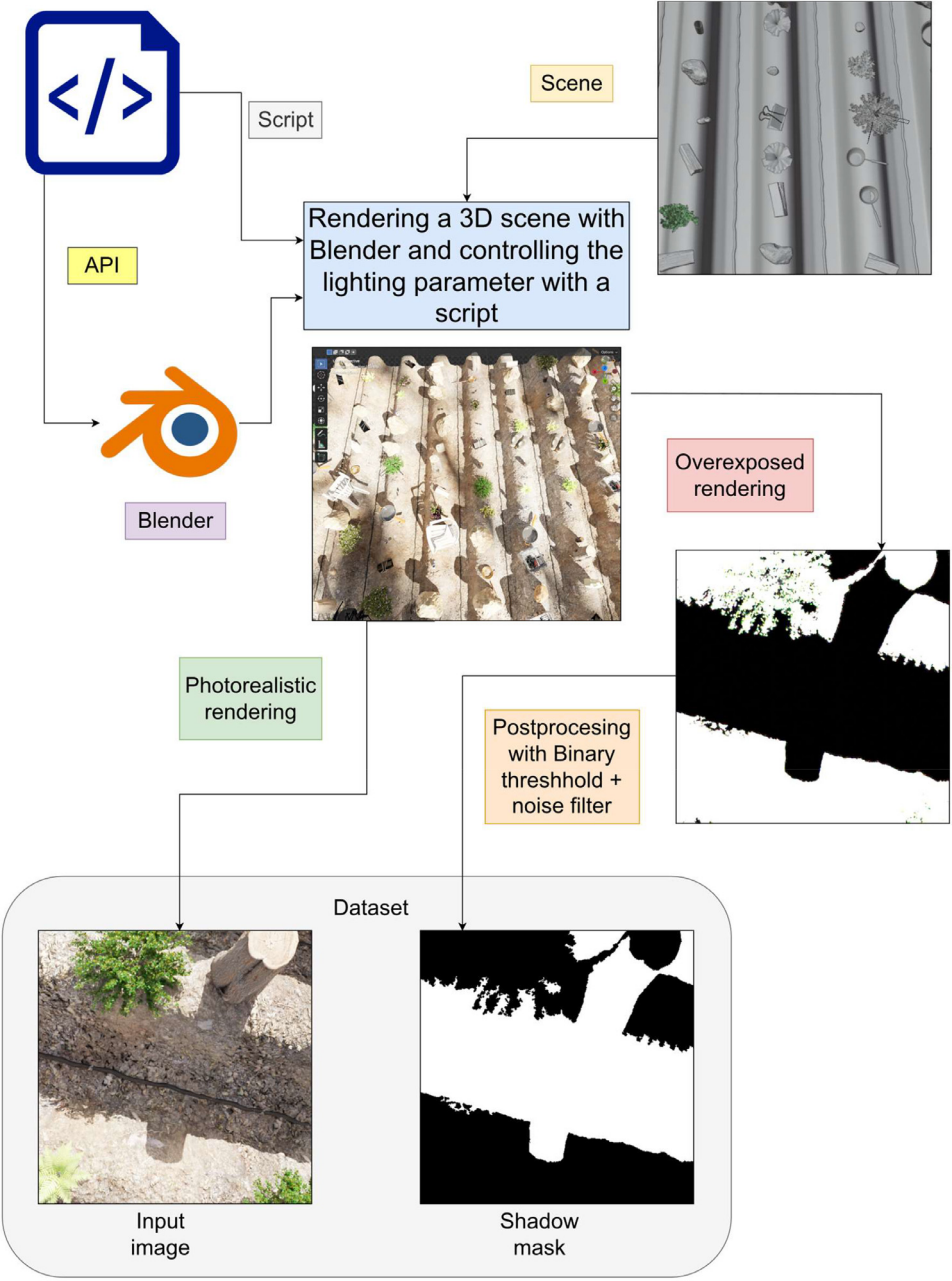
Workflow for the acquisition of synthetic data.

The shadow mask, a critical component in the rendering pipeline, is created by applying a binary threshold to the rendered images. This process involves inverting the image to accentuate shadows rather than sunlight and strategically filtering out small contour areas, aligning with the desired output specifications. The workflow can be succinctly illustrated as depicted in Fig. 4.

In total, 50,000 entries of the dataset are generated using Blender version 3.6.4. Due to our shadow mask workaround, two full renders are required to generate both the image and the shadow mask using an NVidia RTX 4090 in approximately 124 hours. This is achieved with the Cycles renderer, utilizing 1500 samples and a 0.12 adaptive threshold for each image.

## Limitations

Currently, only one procedural virtual scene has been implemented for our dataset, featuring numerous randomly generated parameters. While the procedural approach can enhance diversity, there remains a limit to the variety achievable through randomization alone. And due to the intrinsic attributes of virtual scene design, the dataset is limited to the creation of top-down images specifically within agricultural settings. Given its procedurally generated nature, in instances of very low light environments, certain regions may be erroneously annotated as not being in shadow due to the sun's inclination, despite the actual darkness prevailing in those areas.

## Ethics Statement

The authors declare that the current work does not involve human subjects, animal experiments, or any data collected from social media platforms.

## CRediT authorship contribution statement

**Mengchen Huang:** Conceptualization, Methodology, Software, Validation, Investigation, Data curation, Writing – original draft, Visualization. **Ginés García-Mateos:** Conceptualization, Investigation, Resources, Writing – review & editing, Supervision. **Ruben Fernandez-Beltran:** Conceptualization, Investigation, Resources, Writing – review & editing, Project administration, Funding acquisition.

## Data Availability

AgroSegNet (Original data) (Hugging Face).
